# Risk Factors for Common Bile Duct Stones in Patients with Previous Cholecystectomy: A Multicenter Prospective Proof-of-Concept Study

**DOI:** 10.3390/jcm14134532

**Published:** 2025-06-26

**Authors:** Andrea Lisotti, Thomas Togliani, Graziella Masciangelo, Angelo Bruni, Emilija Rakichevikj, Peter Vilmann, Vincenzo Giorgio Mirante, Pietro Fusaroli

**Affiliations:** 1 Gastroenterology Unit, Hospital of Imola, University of Bologna, 40026 Imola, Italy; masciangelo.lm@gmail.com (G.M.); emilija.rakichevikj@studio.unibo.it (E.R.); pietro.fusaroli@unibo.it (P.F.); 2 Gastroenterology and Digestive Endoscopy Unit, University Hospital Borgo Trento, 37126 Verona, Italy; vg.mirante@gmail.com; 3 Department of Medical and Surgical Sciences, Università di Bologna, 40138 Bologna, Italy; angelo.bruni4@unibo.it; 4 Gastro Unit, Herlev and Gentofte Hospital, 2730 Herlev, Denmark; peter.vilmann@regionh.dk; 5 Department of Clinical Medicine, Faculty of Health Sciences, University of Copenhagen, 1172 Copenhagen, Denmark

**Keywords:** jaundice, sphincterotomy, EUS, ERCP, predictive factors

## Abstract

**Objectives:** Most studies assess risk factors for common bile duct (CBD) stones in patients with gallbladder in situ. We aimed to assess risk factors for CBD stones in patients with previous cholecystectomy in a proof-of-concept study. **Methods:** We enrolled consecutive patients undergoing EUS for suspected symptomatic CBD stones and recorded demographic variables, clinical presentation, liver function tests (LFTs), and transabdominal ultrasound (US) findings. EUS was used as gold standard for CBD stones. Multivariate analysis was used to identify risk factors in the training set; a model was created and tested on the validation set. **Results:** A total of 211 patients (25.6% male; median age, 66 [49–75] years old) were enrolled; 77.7% presented with abdominal pain, 30.3% with hyperbilirubinemia, 26.5% with pancreatitis, and 61.1% with LFT alterations. Ultrasound showed CBD dilation in 37.4% patients. Overall, 96 (45.5%) patients had CBD stones. According to multivariate analysis, male gender (OR 2.54 [1.26–5.09]; *p* = 0.009), age > 63 years (OR 3.06 [1.63–5.72]; *p* < 0.001), LFT alteration (OR 2.62 [1.40–4.91]; *p* = 0.003), and CBD dilation (OR 2.46 [1.31–4.65]; *p* = 0.005) were independently related to CBD stones. A model was created based on the number of risk factors on admission; patients with no risk factor had a 9.5% prevalence of CBD stones; those with one risk factor, 26.7%; two risk factors, 53.2%; three risk factors, 66.7%; and four risk factors, 100%. **Conclusions:** The results of this proof-of-concept study identify male gender, age, LFT alteration, and CBD dilation as risk factors for CBD stones in patients with previous cholecystectomy. An adequate assessment of the pre-test probability will guide patients’ subsequent management.

## 1. Introduction

Common bile duct (CBD) stones are a frequent and clinically significant complication of biliary tract disease. They may lead to a variety of presentations, ranging from mild biliary colic pain to severe conditions such as acute cholangitis and biliary pancreatitis, which carry high morbidity and potential mortality if not promptly diagnosed and managed [1]. Therefore, their timely detection and treatment are crucial for avoiding adverse outcomes and unnecessary procedures. While much of the current literature focuses on the diagnostic approach to CBD stones in patients with intact gallbladders, the prevalence, presentation, and risk profile in post-cholecystectomy patients remain less thoroughly explored.

Following cholecystectomy, anatomical and physiological changes in the biliary tree may influence the formation and detection of CBD stones. These include altered bile flow dynamics, dilation of the CBD, and potential changes in biliary pressure and motility. Additionally, retained or recurrent stones may present with more subtle or delayed clinical signs. Despite these known alterations, most diagnostic algorithms continue to apply criteria derived from pre-cholecystectomy populations, potentially limiting their accuracy and clinical utility in this distinct group [2,3,4,5].

Existing guidelines emphasize the importance of stratifying patients based on their pre-test probability of having CBD stones, using factors such as abnormal liver function tests (LFTs), hyperbilirubinemia, imaging findings like CBD dilation, and the presence of biliary symptoms. However, these criteria were predominantly developed in cohorts with gallbladders in situ. Studies in post-cholecystectomy patients are scarce and often retrospective, making it unclear whether these predictive factors retain the same validity or require adjustment to reflect the altered baseline anatomy [2,3,4,5].

Moreover, post-cholecystectomy patients commonly exhibit mild CBD dilation as a physiological adaptation, which complicates the interpretation of imaging findings such as transabdominal ultrasound (US) results. This makes it critical to re-evaluate the diagnostic utility of non-invasive imaging modalities in this specific population [6,7,8]. Endoscopic ultrasound (EUS), due to its high sensitivity and specificity, has emerged as a valuable diagnostic tool for detecting CBD stones and is often used to confirm or exclude choledocholithiasis, especially in cases with intermediate probability. EUS also offers real-time evaluation of stone size, number, and morphology, which can inform subsequent therapeutic strategies, including the decision to proceed with ERCP in the same session [9,10,11].

Several therapeutic approaches are currently available for the management of common bile duct (CBD) stones, and the choice of modality is influenced by multiple factors, including patient age, anatomical conditions, stone burden, and institutional expertise. ERCP remains the most widely used intervention in adults, offering both diagnostic and therapeutic capabilities in a single session [12]. However, in younger patients or in cases with complex anatomy, the utility of ERCP may be limited due to increased risk of complications or technical challenges. In such scenarios, laparoscopic common bile duct exploration (LCBDE), performed either via a transcystic route or by choledochotomy, has emerged as a safe and effective, minimally invasive alternative, particularly in pediatric and adolescent populations [13]. Additional treatment strategies may include open surgical exploration, especially in patients having undergone failed endoscopic or laparoscopic approaches, as well as percutaneous transhepatic drainage in selected high-risk cases. In asymptomatic patients with small CBD stones, a conservative “watch-and-wait” strategy may be justified. A multidisciplinary and individualized approach remains essential to tailor treatment to each patient’s clinical profile and optimize outcomes [14].

Despite the increasing reliance on EUS in the diagnostic workflow, robust data on its application in post-cholecystectomy patients are limited. Most studies fail to differentiate between patients with and without prior gallbladder removal, creating a gap in the evidence needed for precision medicine in this context. Identifying accurate and independent predictors of CBD stones in patients who have undergone cholecystectomy is therefore essential. Such knowledge would not only enhance the diagnostic algorithm but also guide the appropriate use of advanced imaging modalities and invasive procedures [2,12].

Accordingly, the present multicenter prospective study aimed to systematically assess the risk factors for CBD stones in patients with previous cholecystectomy. By identifying clinical, biochemical, and imaging features independently associated with the presence of CBD stones, we sought to develop a predictive model that can inform clinical decision-making and optimize the diagnostic pathway in this specific and often under-represented population.

## 2. Materials and Methods

### 2.1. Study Design

We conducted a prospective multicenter observational cohort study (level of evidence: III, according to the Oxford Centre for Evidence-Based Medicine criteria), enrolling all consecutive adult patients with previous cholecystectomy admitted for suspected symptomatic CBD stones from January 2019 to December 2020. Patients referred to the Hospital of Mantova were included in the training set, and patients referred to the Hospital of Imola were included in the validation set. Patients were excluded if they had a previous diagnosis of CBD stones; acute cholangitis requiring urgent ERCP; incomplete medical records; previous ERCP or percutaneous biliary interventions; a history of upper gastrointestinal, hepatic, biliary, or pancreatic surgery (other than cholecystectomy); a history of hepatic, pancreatic, or biliary neoplasia; cholestatic chronic liver disease; or liver cirrhosis. The study was conducted in accordance with the principles of the Declaration of Helsinki (revision of Edinburgh, 2000). Prior to inclusion in the study, all patients provided written informed consent to the diagnostic procedures and for anonymous review of clinical data for research purposes. The local Institutional Review Board (Ethics Committee—Hospital of Imola: “Comitato Etico Indipendente, Azienda USL di Imola) approved the observational design of the study on 24 October 2018 (study code N° 332-2018-OSS-AUSLIM). Each local IRB then approved the protocol. This was a non-interventional, observational study; therefore, registration in a clinical trials registry was not required under national and institutional guidelines.

### 2.2. Study Population and Eligibility Criteria

Eligible participants were adult patients (≥18 years) with a prior cholecystectomy, referred for evaluation of suspected symptomatic CBD stones. Suspicion was based on clinical presentation (e.g., right upper-quadrant pain, jaundice, or acute pancreatitis), biochemical abnormalities, or suggestive imaging findings. Patients were divided into a training set (Hospital of Mantova) and a validation set (Hospital of Imola) to allow for model testing in an independent cohort. Exclusion criteria included (a) previous diagnosis of CBD stones or prior biliary stone extraction (via ERCP or percutaneous drainage); (b) acute cholangitis requiring emergent ERCP; (c) incomplete clinical or laboratory data; (d) history of hepatic, pancreatic, or biliary malignancy; (e) history of other gastrointestinal surgeries beyond cholecystectomy; (f) known chronic liver diseases (e.g., primary sclerosing cholangitis or cirrhosis); and (g) inability to provide informed consent.

### 2.3. Clinical and Laboratory Assessment

Demographic data, clinical symptoms, laboratory parameters, and imaging findings were prospectively recorded at the time of admission. Liver function tests (LFTs) included alanine aminotransferase (ALT), aspartate aminotransferase (AST), alkaline phosphatase (ALP), and total bilirubin.

### 2.4. Imaging Modalities and Diagnostic Approach

All patients underwent transabdominal ultrasound (US) as the initial imaging modality. CBD diameter and the presence of stones or sludge were documented. Subsequently, endoscopic ultrasound (EUS) was performed by expert endosonographers using standard linear-array echoendoscopes. EUS was performed in the left-lateral decubitus; moderate to deep sedation was administered based on physicians’ preferences. EUS served as the gold standard for CBD stone diagnosis, enabling direct visualization and characterization of stones in terms of number, size, and location.

### 2.5. Definitions and Outcome Measures

The aim of this study was to identify risk factors for CBD stones in patients with previous cholecystectomy, using EUS as the gold-standard reference for diagnosis. The primary outcome of the study was the presence of CBD stone according to EUS, while secondary outcomes were the identification of independent predictors of CBD stones and validation of a stratification model. We analyzed demographic variables, clinical presentations, liver function tests (LFTs), and transabdominal ultrasound (US) findings to identify independent variables correlated with the presence of CBD stones and develop a model to stratify the pre-test risk of CBD stones. Hyperbilirubinemia was defined as the presence of a serum bilirubin level ≥ 1.3 mg/dL [2]. LFT alteration was defined as the presence of increased alkaline phosphatase (>125 IU/mL) or transaminase (>1.5) beyond the upper normal limit [2]. CBD dilation was assessed with transabdominal US; CBD size was included as a continuous variable. Recent cholecystectomy was defined as surgery performed within 6 months before enrollment.

### 2.6. Statistical Analysis

Continuous variables were reported as median [interquartile range, IQR] or mean ± standard deviation according to their distribution tested with the Shapiro–Wilk test and the visual analysis of the distribution histograms and compared using the Mann–Whitney test or *t*-test when appropriate; categorical variables were reported as numbers (percentages) and compared using Fisher’s exact test. Receiver operator characteristic (ROC) curve analysis was used to identify the best cut-off values for CBD size and age using Youden’s index. Univariate and multivariate analyses were conducted on the training set to identify independent risk factors for CBD stones. Univariate logistic regression was used to identify risk factors for CBD stones. Variables with *p* values < 0.05 in the univariate analysis were included in the multivariate logistic regression model to determine independent risk factors. Odds ratios (ORs) and 95% confidence intervals (CIs) were calculated. A model was developed based on multivariate analysis results and tested on the validation set. A post hoc sample-size analysis was performed using the Events Per Variable (EPV)” rule. MedCalc Statistical Software version 19 (MedCalc Software, Ostend, Belgium; https://www.medcalc.org; 2019 (accessed on 25 April 2023)) was used.

## 3. Results

### 3.1. Patients’ Characteristics

**Figure 1:**
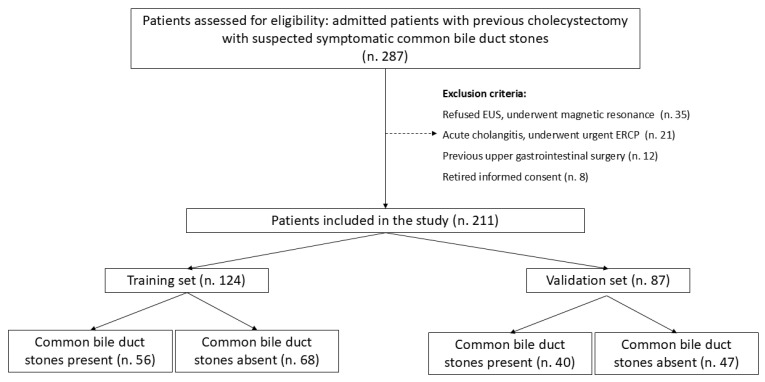
Study flow chart.

Overall, 211 patients (male 54, 25.6%) were enrolled in the study. The median age was 66 [49–75] years. Figure 1 shows a flow chart of the study.

**Figure 2:**
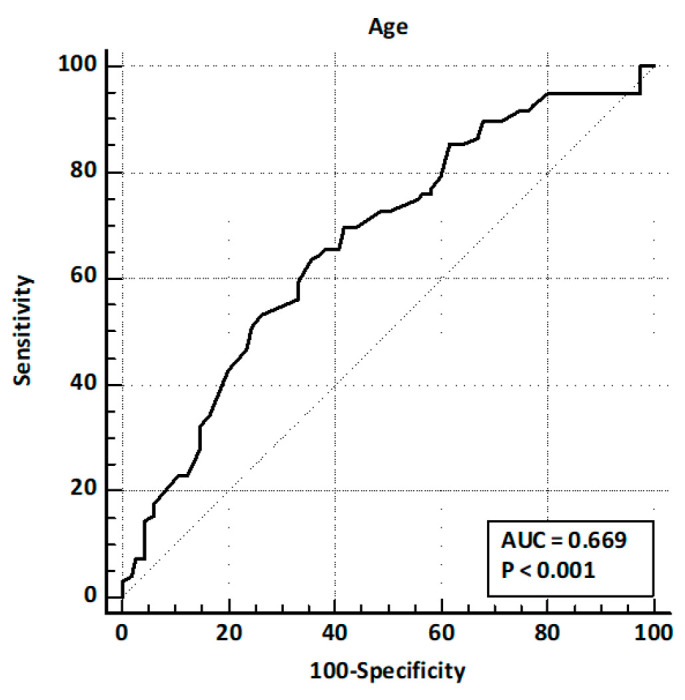
ROC curve analysis for age.

**Figure 3:**
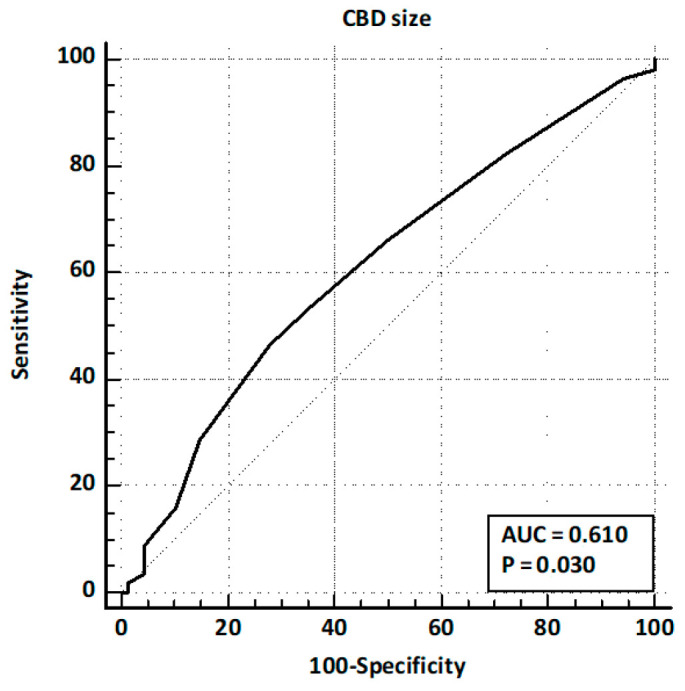
ROC curve analysis for CBD size.

Patients presented after a median of 32 months [5–60] after cholecystectomy. Of these, 164 (77.7%) presented with abdominal pain, 56 (26.5%) with acute pancreatitis, and 64 (30.3%) with hyperbilirubinemia. LFTs were altered in 129 (61.1%) patients, and CBD dilation was observed in 79 (37.4%) cases. EUS identified CBD stones in 96 patients (45.5%). The comparison of baseline characteristics between training and validation sets showed a significantly higher prevalence of hyperbilirubinemia and LFT alterations in the validation set, while other baseline characteristics and prevalence of CBD stones were similar (Table 1). ROC curve analysis identified age > 63 years and CBD size > 8 mm as the best cut-off values (Figure 2 and Figure 3).

Risk factors for CBD stones. Univariate analysis was conducted to identify factors associated with the presence of CBD stones on EUS (Table 2). The analysis revealed that male gender (OR 1.91 [1.02–3.57]; *p* = 0.04), age > 63 years (OR 3.22 [1.82–5.71]; *p* < 0.001), hyperbilirubinemia (OR 1.86 [1.03–3.37]; *p* = 0.04), LFT alterations (OR 2.15 [1.21–3.81]; *p* < 0.001), and CBD dilation (OR 2.71 [1.53–4.81]; *p* < 0.001) were significantly associated with the presence of CBD stones. Abdominal pain (OR 0.93 [0.49–1.79]; *p* = 0.84) and acute pancreatitis on presentation (OR 0.95 [0.52–1.76]; *p* = 0.88), as well as detection of CBD stones by US (OR 18.2 [0.21–126.3]; *p* = 0.66), were not. Time elapsed from cholecystectomy to suspicion of a symptomatic CBD stone was not corelated, nor was assessment as a continuous variable (OR 1.02 [0.97–1.12]; *p* = 0.32) or as recent (<6 months) cholecystectomy (OR 0.62 [0.21–3.28]; *p* = 0.64).

**Figure 4:**
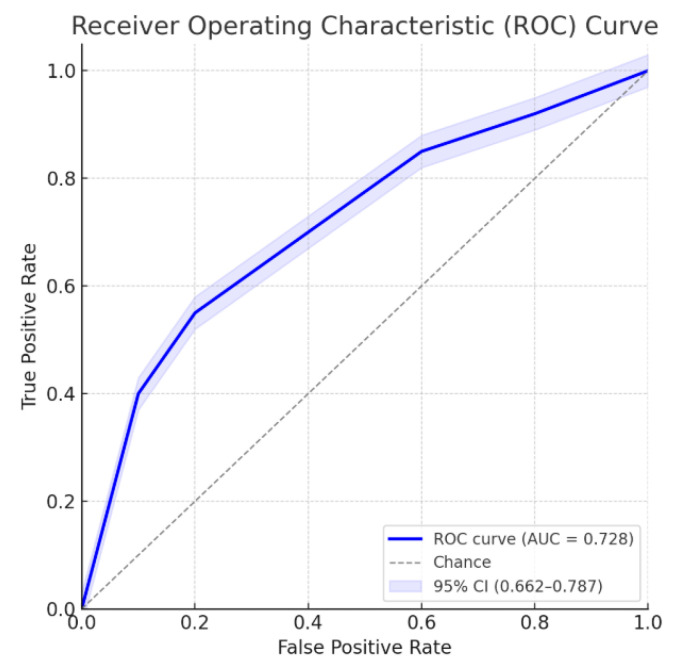
ROC curve for the multivariate model. Area under the ROC curve: 0.728; 95% confidence interval: 0.662–0.787.

**Figure 5:**
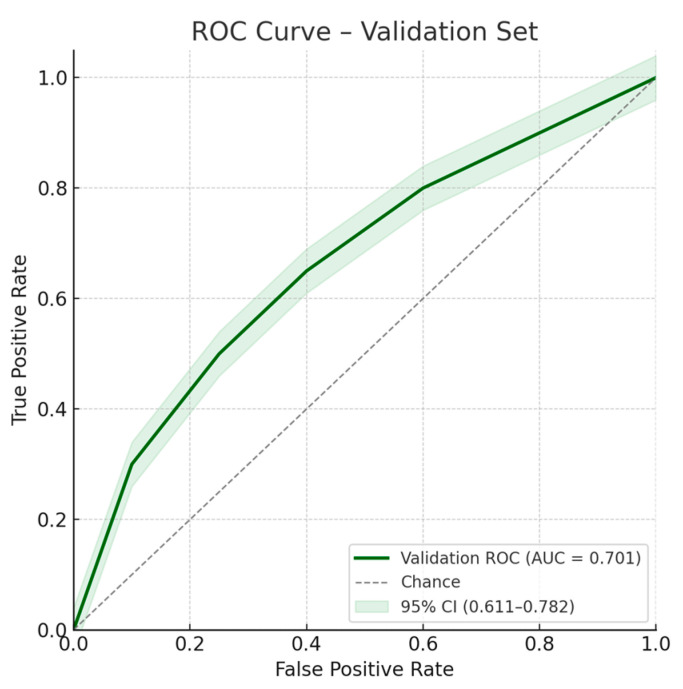
ROC curve for the multivariate model on the validation set. Area under the ROC curve: 0.701; 95% confidence interval: 0.611–0.782.

Multivariate analysis, performed with the stepwise regression model, identified male gender (OR 2.54 [1.26–5.09]; *p* = 0.009), age > 63 years (OR 3.06 [1.63–5.72]; *p* < 0.001), LFT alterations (OR 2.62 [1.40–4.91]; *p* = 0.003), and CBD dilation (OR 2.46 [1.31–4.65]; *p* = 0.005) as independent risk factors for the presence of CBD stones. Similar estimates have been observed with the enter regression model, as reported in Table 2. ROC curves for the model on the training and validation sets are shown in Figure 4 and Figure 5, respectively.

### 3.2. Model for Suspected CBD Stone 

**Figure 6:**
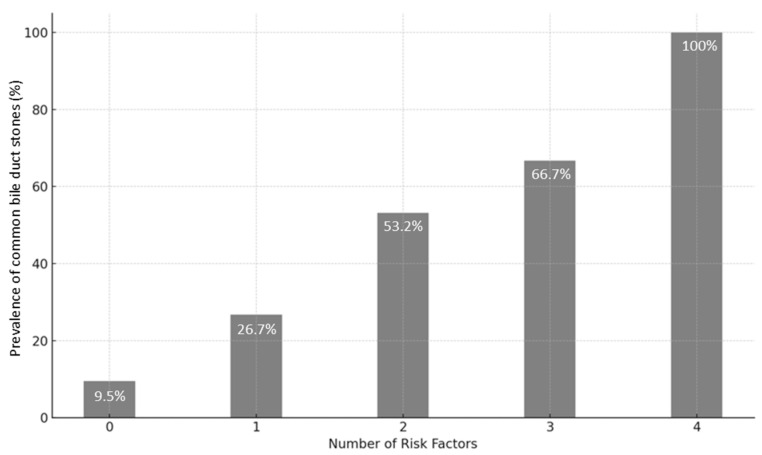
Performance of predictive model assessed on the validation set.

Based on the results of the multivariate analysis, a model was developed to stratify the risk of CBD stones. Since the ORs were similar among all the independent risk factors (namely, male gender, age > 63 years, LFT alteration, and CBD dilation), we developed a simplified model based on the number of risk factors present on admission. The incidence of CBD stones, tested on the validation set, is reported in Figure 1. In detail, patients with no risk factor had a 9.5% prevalence of CBD stones according to EUS; the prevalence of CBD stones increased to 26.7% in patients with one risk factor, 53.2% when two risk factors were present, 66.7% in patients with three risk factors, and 100% in patients with all four risk factors (Figure 6).

## 4. Discussion

This prospective multicenter study investigated the clinical, biochemical, and imaging predictors of common bile duct (CBD) stones in patients with a history of cholecystectomy. Our results demonstrate that male gender, age > 63 years, liver function test (LFT) alterations, and CBD dilation on ultrasound are independent risk factors for the presence of CBD stones, as confirmed by endoscopic ultrasound (EUS) [9,10,11,12]. Based on these variables, we developed a simplified scoring model that effectively stratifies patients by risk of CBD stones and may assist clinicians in guiding further diagnostic and therapeutic strategies [2,3,4].

These findings carry several important implications for post-cholecystectomy patients, a group frequently encountered in clinical practice but often under-represented in diagnostic algorithms. While existing guidelines predominantly focus on populations with gallbladders in situ, our study highlights the necessity of adapting risk stratification tools to reflect the altered anatomy and physiology following cholecystectomy, including the common occurrence of mild baseline CBD dilation and potentially different symptom profiles. In detail, our findings align with existing literature by confirming age, LFT alterations, and CBD dilation as key predictors for CBD stones while also highlighting male gender as a novel independent risk factor in post-cholecystectomy patients, adding depth to the understanding of stone recurrence in this population [1,12]. Surprisingly, the identification of CBD stones by US was not correlated with the presence of stones according to multivariate analysis due to the low diagnostic accuracy; indeed, the optimal (up to 100%) specificity of US in diagnosing CBD stones was counterbalanced by a very low (<50%) sensitivity.

The identification of four independent risk factors, namely male gender, age > 63 years, LFT alterations, and CBD dilation on US, could have several clinical implications. Indeed, our model showed a clear correlation between the number of risk factors and the likelihood of CBD stones: from 9.5% in patients with no risk factors up to 100% in those with all four. This linear gradient underscores the potential utility of a stepwise pre-test probability approach to optimize clinical decision-making. In high-probability cases (≥3 risk factors), a one-step diagnostic and therapeutic procedure using same-session EUS and ERCP could be justified, reducing unnecessary imaging such as MRCP, shortening time to treatment, and improving patient flow through endoscopy units. For example, we hypothesized that patients presenting with three or more risk factors can be directly scheduled for same-session EUS and ERCP based on the high pre-test probability of CBD stones (66.7% and 100%, respectively).

Although hyperbilirubinemia was significantly associated with the presence of CBD stones in the univariate analysis (OR 1.86, *p* = 0.04), it did not retain independent predictive value in the multivariate model. This finding likely reflects statistical collinearity between hyperbilirubinemia and other more robust indicators of biliary obstruction, such as LFT alterations and CBD dilation. Furthermore, our study excluded patients with acute cholangitis, who typically presents with jaundice. The exclusion of these cases may have reduced the overall variability and strength of association between bilirubin levels and CBD stones in our cohort. These considerations may explain why hyperbilirubinemia, despite its clinical relevance, did not emerge as an independent predictor in the adjusted model.

The finding that male gender was an independent predictor of CBD stones in this population adds a novel dimension to the current literature, which often underestimates the influence of sex-based differences in biliary pathology. Although, traditionally, female gender is more associated with gallstone formation, our results suggest that, post-cholecystectomy, the risk landscape may shift, potentially due to changes in bile composition, hormone metabolism, or delayed presentation among male patients [13,14,15,16,17].

Interestingly, while abdominal pain and acute pancreatitis were frequent presentations, they were not significantly associated with the presence of CBD stones in either univariate or multivariate analysis. This suggests that symptomatology alone may not be sufficient to discriminate between patients with and without CBD stones, particularly in a post-cholecystectomy setting. Similarly, CBD stones identified by transabdominal ultrasound had low sensitivity, despite high specificity, reinforcing the known limitations of ultrasound in this diagnostic scenario and the value of EUS for definitive diagnosis [18,19,20].

Periampullary diverticulum (PD) and angulation of the distal common bile duct (ADCBD) are anatomical factors known to contribute to biliary stasis and have been implicated in the pathogenesis of common bile duct stones. In our cohort, PD was observed in 7.1% of patients, while ADCBD was present in 6.3% of patients who underwent ERCP after confirmation of CBD stones. However, these findings were not incorporated into the predictive model for several reasons. Firstly, PD was an endoscopic finding (during EUS in this study) and, thus, cannot be used as a criterion for selecting patients for EUS examination. Secondly, ADCBD is typically identified during ERCP after biliary cannulation or on magnetic resonance cholangiopancreatography (MRCP), meaning these data were only available for patients with already confirmed CBD stones in this study. Consequently, ADCBD and PD do not serve as practical predictive factors in the initial diagnostic pathway and were, therefore, excluded from the multivariate analysis. These considerations highlight the challenges of integrating into early predictive models anatomical variations that are identifiable only after the confirmation of stones [12,13,14].

This combined diagnostic and therapeutic approach of performing EUS and ERCP in the same session is crucial for optimizing patient outcomes and spare time in the endoscopic schedule and in reducing the carbon footprint of the procedures [21,22]. Furthermore, it lowers anesthesiologic risks by minimizing exposure to repeated sedations, especially in elderly or high-risk patients [23]. The ability to address both diagnosis and treatment concurrently ensures more efficient care and reduces the likelihood of recurrent biliary events, which are common in patients with untreated CBD stones [24].

Moreover, it is also noteworthy that patients in the validation set had a significantly higher prevalence of hyperbilirubinemia and LFT alterations compared to those in the training set. This reinforces the importance of these clinical signs as strong indicators of CBD stones in patients with previous cholecystectomy. Although abdominal pain and acute pancreatitis did not emerge as statistically significant predictors, they were frequent clinical presentations in our cohort, suggesting their continued relevance in the clinical evaluation of these patients, even if they are not independently associated with the presence of stones.

A major strength of this study is the prospective design, which enhances the generalizability of the results; it has been emphasized in the most recent guidelines that most studies in this field have poor methodological quality. Additionally, we used an independent validation set to test our model. On the other hand, our study presents several limitations. First of all, the relatively small size of both populations may affect the statistical power of the logistic regression analyses; even if this study lacks an a priori sample size calculation, the sample size for this prospective study was based on the number of outcome events needed for multivariate logistic regression analysis. According to the EPV rule, a minimum of 10 outcome events are required per independent variable. With four predictors in our final model and 96 patients with confirmed CBD stones (events), our study exceeded the minimum requirement of 40 events, ensuring adequate statistical power and model stability. Although a sample size calculation was not performed a priori, the post hoc event-based analysis supports the validity of our multivariable approach. Moreover, the exclusion of patients presenting with acute cholangitis requiring urgent ERCP could have impacted on some of the observed results; surprisingly, we failed to identify the presence of hyperbilirubinemia as an independent risk factor. Finally, we excluded all patients with previous CBD interventions (ERCP, percutaneous); with previous surgery; and with hepatic, pancreatic, or biliary neoplasia in order to enroll a homogeneous population. We acknowledge that this tight selection does not reflect the real-life setting of patients with suspected CBD stones, who usually have several comorbidities and medical history. Finally, the design of this study does not allow us to assess possible risk factors related to previous surgery, such as between the cholecystectomy and the enrollment and the possible difficulties encountered during surgery.

**Figure 7:**
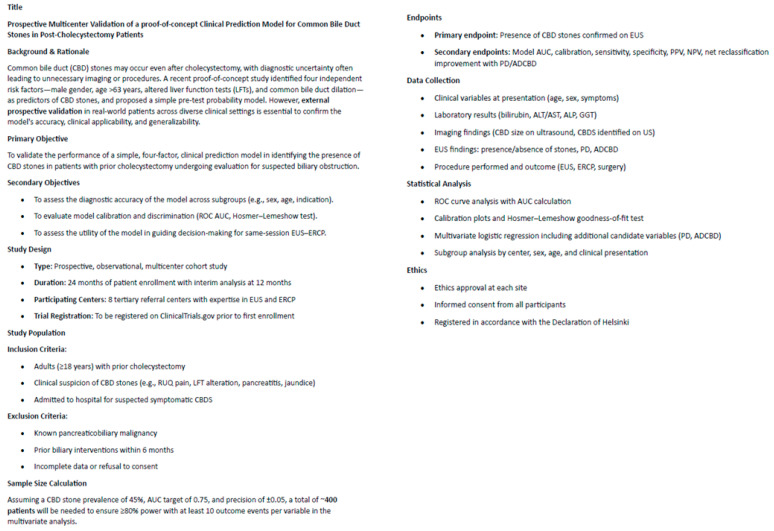
Synopsis of a prospective confirmatory study.

Several methodological issues must be kept in mind during the interpretation of the results of this proof-of-concept study. In detail, the lack of a predefined sample size calculation, the 2-year study period, and the single-center nature of the validation cohort limit the statistical power of the results and model reliability. Since all the authors agreed that confirmation by a large multicenter prospective study is required, a prospective study has already been planned, as demonstrated by the synopsis shown in Figure 7.

Future research should aim to validate our model in larger and different populations and explore whether additional clinical or laboratory markers could further refine the performance of the model. In particular, we suggest an external validation of this model in larger, multicenter, and more diverse populations, including those with prior CBD interventions or comorbidities, as well as assessment of the role of additional biomarkers (e.g., inflammatory markers or bile biochemistry) to enhance predictive accuracy, investigation of the cost-effectiveness and patient outcomes of using this risk-based model to guide same-session EUS and ERCP, the exploration of gender-specific differences in CBD stone recurrence and pathophysiology after cholecystectomy [25,26,27,28,29,30,31], and the possible application of novel approaches to treat benign and malignant biliary conditions [32,33].

## 5. Conclusions

In conclusion, this study provides insightful observations for the management of symptomatic patients admitted for suspected CBD stones. The identification of risk factors for CBD stones in patients with previous cholecystectomy and the development of a practical model may support clinical decision-making in this field, providing timely interventions in high-risk patients and sparing resources in patients with low pre-test probability of having CBD stones. The results of this proof-of-concept study must be validated in larger and independent studies.

This study supports a more tailored diagnostic strategy for post-cholecystectomy patients with suspected CBD stones. The proposed model allows for personalized risk assessment, helping to avoid unnecessary procedures in low-risk patients while expediting treatment in high-risk individuals. Integration of this approach into clinical pathways could improve resource allocation, reduce diagnostic delays, and enhance patient safety.

## Figures and Tables

**Table 1:** Baseline characteristics.

Characteristic	Total (No. 211)	Training Set (No. 124)	Validation Set (No. 87)	*p* Value ^‡^
Demographics				
Gender (male), No. (%)	54 (25.6%)	33 (26.6%)	21 (24.1%)	0.75 ^a^
Age (years), median [IQR]	66 [49–75]	65.5 [49–75]	68 [49.25–76]	0.78 ^b^
Time from cholecystectomy (months)	32 [5–60]	20 [6–55]	36 [9–60]	0.29 ^b^
Clinical presentation				
Abdominal pain, No. (%)	164 (77.7%)	96 (77.4%)	68 (78.2%)	1.00 ^a^
Acute pancreatitis, No. (%)	56 (26.5%)	28 (22.6%)	28 (32.2%)	0.15 ^a^
Hyperbilirubinemia, No. (%)	64 (30.3%)	27 (21.8%)	37 (42.5%)	0.001 ^a^
Biochemistry				
Liver function test alteration, No. (%)	129 (61.1%)	66 (53.2%)	63 (72.4%)	0.01 ^a^
Imaging findings on US				
Common bile duct dilation, No. (%)	79 (37.4%)	45 (36.3%)	34 (39.1%)	0.77 ^a^
Common bile duct stones, No. (%)	12 (5.7%)	8 (6.5%)	4 (4.6%)	0.88 ^a^
EUS results				
Common bile duct stones, No. (%)	96 (45.5%)	56 (45.2%)	40 (46.0%)	1.00 ^a^
Periampullary diverticulum, No. (%)	15 (7.1%)	10 (8.1%)	5 (5.7%)	0.86 ^a^

Abbreviations: No.—number; IQR—interquartile range; US—ultrasound. **^‡^** *p* value for the comparison between baseline characteristics of the training set and validation set. ^a^ Fisher’s exact test. ^b^ Mann–Whitney test.

**Table 2:** Risk factors for presence of common bile duct stones on EUS.

	Odds Ratio [95% C.I.]	*p* Value	Odds Ratio [95% C.I.]	*p* Value	Odds Ratio [95% C.I.]	*p* Value
	Univariate analysis		Multivariate analysis (stepwise)		Multivariate analysis (Enter)	
Gender (male)	1.91 [1.02–3.57]	0.04	2.54 [1.26–5.09]	0.009	2.53 [1.26–5.09]	0.009
Age (>63 years)	3.22 [1.82–5.71]	<0.001	3.06 [1.63–5.72]	<0.001	3.04 [1.62–5.70]	0.0005
Time from cholecystectomy	1.02 [0.97–1.12]	0.32	---	---	---	---
Recent cholecystectomy	0.62 [0.21–3.28]	0.64	---	---	---	---
Abdominal pain	0.93 [0.49–1.79]	0.84	---	---	---	---
Acute pancreatitis	0.95 [0.52–1.76]	0.88	---	---	---	---
Hyperbilirubinemia	1.86 [1.03–3.37]	0.04	NS *	NS *	1.54 [0.77–3.10]	0.22
Alkaline phosphatase > 125 U/L	1.78 [0.94–3.22]	0.11	---	---	---	---
AST > 1.5 upper limit normal	1.45 [0.60–2.45]	0.54	---	---	---	---
ALT > 1.5 upper limit normal	1.56 [0.55–2.02]	0.24	---	---	---	---
LFTs alteration	2.15 [1.21–3.81]	<0.001	2.62 [1.40–4.91]	0.003	2.26 [1.15–4.42]	0.002
CBD dilation on US	2.71 [1.53–4.81]	<0.001	2.46 [1.31–4.65]	0.005	2.53 [1.34–4.81]	0.005
CBD stones on US	18.2 [0.21–126.3]	0.66	---	---	---	---

Abbreviations: LFTs—liver function tests; CBD—common bile duct; US—ultrasound; 95% C.I.—95% confidence interval; NS—not statistically significant. * A stepwise multivariate model was used; thus, variables found to be not statistically significant were excluded from the model, and no confidence interval or *p* value could be provided.

## Data Availability

The data presented in this study are available upon request from the corresponding author due to privacy and regulatory issues.

## Abbreviations

The following abbreviations are used in this manuscript:

CBD	Common Bile Duct
EST	Endoscopic Biliary Sphincterotomy
EUS	Endoscopic Ultrasound
ERCP	Endoscopic Retrograde Cholangiopancreatography
PAD	Periampullary Diverticulum
LFTs	Liver Function Tests
OR	Odds Ratio
CI	Confidence Interval
IQR	Interquartile Range
TBIL	Total Bilirubin
WBC	White Blood Cell (count)
MRCP	Magnetic Resonance Cholangiopancreatography
